# Which body functions and activities matter for stroke patients? Study protocol for best–Worst scalings to value core elements of the International Classification of Functioning, Disability and Health

**DOI:** 10.1371/journal.pone.0295267

**Published:** 2023-12-07

**Authors:** Christin Juhnke, Axel Christian Mühlbacher

**Affiliations:** 1 Health Economics and Health Care Management, Hochschule Neubrandenburg, Neubrandenburg, Germany; 2 Duke Department of Population Health Sciences and Duke Global Health Institute, Duke University, Durham, North Carolina, United States of America; Cairo University, EGYPT

## Abstract

**Background:**

Stroke is a common, serious, and disabling healthcare problem with increasing incidence and prevalence. Following a stroke, identifying the factors associated with decisions about rehabilitation interventions is important to assess rehabilitation after stroke. The aim is to guide clinical staff to make patient-centered decisions. Fundamentally, decision makers cannot draw on evidence to consider the relevance of distinct functions and activities from the patient’s perspective. Until now, outcomes of rehabilitation are generally categorized using the International Classification of Functioning, Disability and Health (ICF). This can be seen as a conceptual basis for the assessment of health and disability. Since the ICF does not distinguish importance between these aspects there is a need to value the most important clinical factors as well as related activities from a patients and public perspective to help guide therapists in effectively designing post-acute rehabilitation care for individuals following stroke. The research question is which ICF body functions and activities are of value to stroke patients? Which trade-offs are patients willing to make within the core elements? Health preference research (HPR) answers the need to develop additional preference weights for certain ICF dimensions. Patient preference information (PPI) values health conditions based on the ICF from a patient perspective.

**Methods:**

In this study we conduct three best–worst scaling (BWS) experiments to value body function and activities from patients’ and public perspective. Out of all ICF dimensions this research covers health conditions relevant to stroke patients in terms of body function, perception, and activities of daily living. Stroke patients as well as members of the general population will be recruited to participate in the online BWS surveys. Fractional, efficient designs are applied regarding the survey design. Conditional and multinominal logit analyses will be used as the main analysis method, with the best–worst count analysis as a secondary analysis. The survey is being piloted prior to commencing the process of data collection. Results are expected by the autumn of 2023.

**Discussion:**

The research will add to the current literature on clinical decision-making in stroke rehabilitation and the value of certain body functions as well as related activities in neurorehabilitation. Moreover, the study will show whether body functions and activities that are currently equally weighted in international guidelines are also equally important from the point of view of those affected, or whether there are disconcordances in terms of differences between public judgements and patients’ preferences.

## 1. Introduction

### Clinical background

Stroke is a common, serious, and disabling healthcare problem [1]. In most European countries, stroke is the second or third most common cause of death and one of the main causes of acquired adult disability [2, 3]. While the prevalence of stroke-related burden is expected to increase over the next two decades most patients with stroke will survive the initial illness. They require resources to get back to the community after acute hospital care. The greatest health effect is usually caused by the long-term consequences for patients and their families. The more severe the stroke, the significantly larger the economic impact on healthcare services [4, 5]. Stroke-related disabilities ("neuro-disabilities") are increasing significantly worldwide. Hence, ensuring the prompt rehabilitation provision includes decisions regarding what level of care and which services are essential for stroke survivors in a post-acute phase. Therefore, decision making requires a comprehensive knowledge of health status, realistic discharge goals, and a full consideration of desired rehabilitation goals, including functional independence and psychosocial well-being [6]. However, decision by patients and physicians are not always congruent [7, 8]. Through medical and adequate neurorehabilitative treatment, the disabilities can be sustainably reduced, and patients can regain more independence in their daily lives. This requires more capacities of physiotherapists, occupational therapists, or neuropsychologists, which are currently not available in the healthcare system [9].

### Decisional background

The value drivers of neurorehabilitation are largely unknown, especially from the patient’s perspective. Patients are increasingly exposed to a variety of unknown therapies. Accordingly, there is limited evidence on the characteristics of patient acceptance regarding neurorehabilitation following stroke. Little is known about how patients make decisions for desired outcomes of rehabilitation care, especially after stroke. A multitude of factors influence the decisions: the clinical prognosis, goals for rehabilitation [10], social attributes [11] and desired activities of daily living [12] are important in deciding rehabilitation needs.

Decision-making, especially concerning impaired clinical functions addressed in rehabilitation of stroke patients, is currently guided by the clinical perspective with no specific tools to guide the process. Hence, deciding and assessing rehabilitation for patients with high levels of disabilities is subject to several ethical dilemmas and subjective biases including the value of the clinicians about treating severely disabled patients [4, 13], overestimating or underestimating the impact of stroke on the patient’s health and their expected quality of life [14], fear of litigation and knowledge of the likely outcomes of various interventions [15], which may all contribute to variation in decision-making.

The decision and evaluation of rehabilitation for patients with neuro-disabilities is therefore subject to several manifold dilemmas and subjective biases, including clinicians’ assessments for the best treatments for patients [16, 17], overestimation or underestimation of the impact of stroke on the patient’s health status, quality of life [14] and expected activities, all of which may contribute to differential decision making [16].

**Fig 1:**
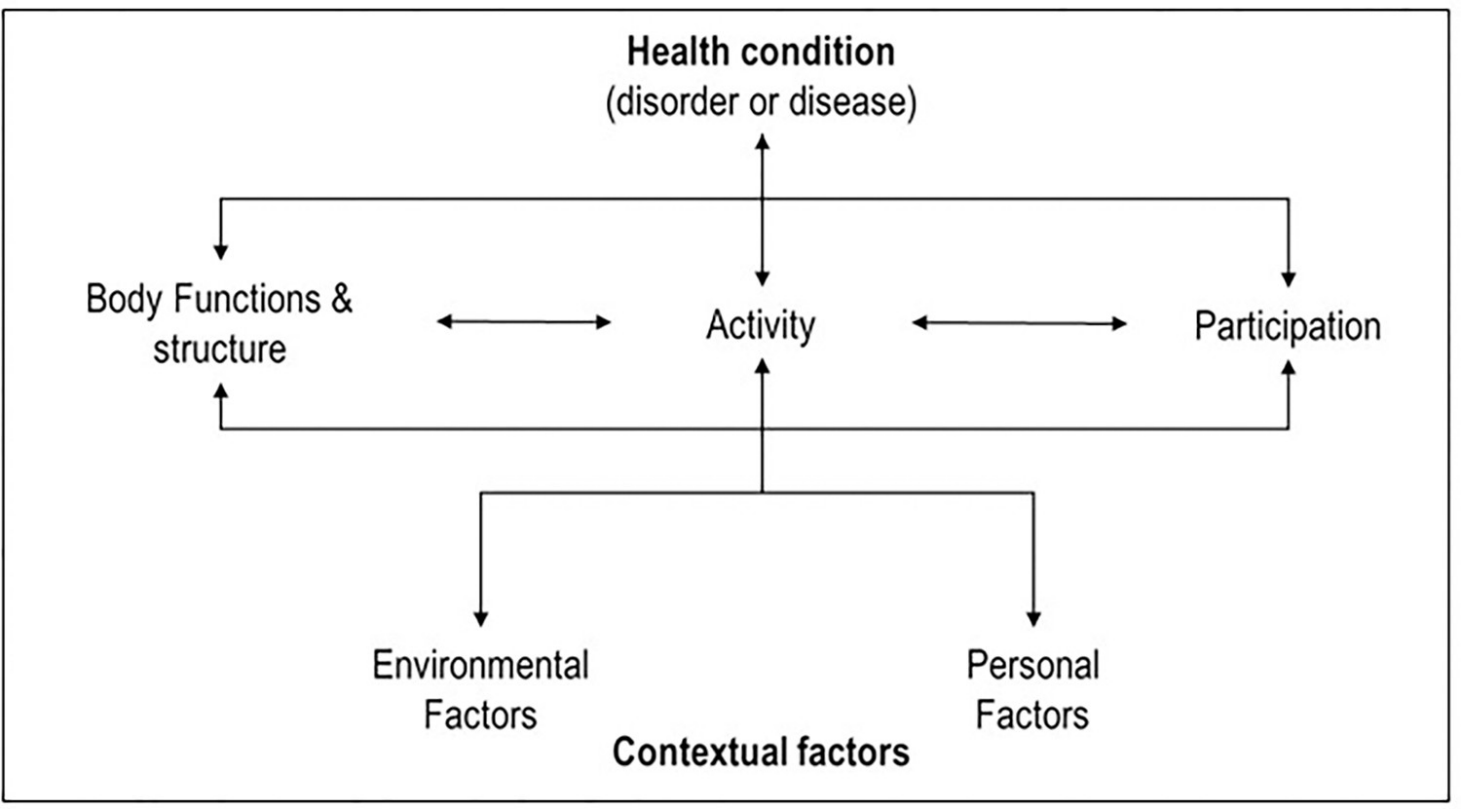
Model of physical disabilities according to ICF [19].

The decisions for rehabilitation of severe stroke patients must not be based on known prognostic indicators or rehabilitation requirements alone, but on patient-relevant characteristics [16, 18]. To assess the degree of impairment in clinical functions and activities following stroke, most clinicians use the universal International Classification of Functioning, Disability and Health (ICF) [19]. Within the ICF rehabilitation practitioners can rely for the first on a worldwide accepted model providing a universal language for the description and classification of functioning. The framework is used to describe human functioning and disability. The overarching concept of the ICF is functional health (= functioning), which is mapped in its components of body function and structure, and activity and participation (Fig 1) [19].

The ICF is divided into different subchapters and subcategories within each component. For every function and activity, the ICF gives a generally valid, very clinical definition. But the question arises of how to assign a value to these functions.

The ICF is a framework on which tools for measuring or ‘assessing’ individual functioning may be based, and to which they can be mapped. The broad framework puts assessment in context and provides the focus for selecting relevant aspects of functioning and disability for assessment. The credo of the ICF is: "From body functions arise activities" [19]. Since the value of a body function is derived from associated activities, this study will conduct an extra choice experiment on the activities to subsequently link this to the functions and measure their value.

The ICF is shown to be an essential tool for identifying and measuring efficacy and effectiveness of rehabilitation services, both through functional profiling and intervention targeting [20]. However, all functions and activities within the classification are equally important.

## 2. Objectives

It is unclear whether patients consider all ICF factors collectively or prioritize certain factors in deciding rehabilitation treatments. Assessing key clinical factors and related activities from both patients and public perspectives is crucial for guiding therapists and shaping post-stroke rehabilitation programs. The effectiveness of interventions relies on clinical outcomes, patient self-assessment, and perceived effort, yet there is limited empirical evidence on which neurorehabilitation attributes patients consider important [21].

This study aims to understand the trade-offs stroke patients are willing to make for neurorehabilitation and identify pivotal attributes influencing their treatment adherence. It will explore heterogenous preferences among patients and use personalized information for a person-centered approach.

Understanding how patients and citizens value different body functions in rehabilitation decision-making is vital [8]. The research aims to identify critical clinical factors and their significance in decision-making for robot-assisted interventions, incorporating perspectives from both patients and the general population.

## 3. Descriptive framework

Preference evidence is a series of human behaviors. Different elicitation tasks can be used to observe behavior. Preference elicitation involves setting up tasks that confront subject with assigned objects around which their preferences can be expressed and observed.

In this study, we will elicit patients’ willingness to accept tradeoffs among treatment attributes that were derived from the International classification of functioning, disability and health [19].

For the experiment on body functions the basis is ICFs chapter 7 “Neuromusculoskeletal and movement related functions” of the body function section. The first branching level ("second level classifications") categorized in three sub-chapters is used as attributes and encompasses twelve aspects. The general scale of assessment characteristics with negative scale of the ICF are used as levels. The levels reflect the labeling of the extent or magnitude of the impairment. The characteristics "not specified" and "not applicable" of the ICF scale are not considered. This results in three levels (Table 1).

**Table 1:** List of attributes.

Experiments			
	Body functions	Neglect	Activities
**Attributes**	Joint mobility	Visual-spatial perception	Learning & knowledge application: Conscious sensory perceptions
	Joint stability	Orientation to time	Learning &knowledge application: elementary learning
	Mobility of bones	Orientation to place	Learning &knowledge application: Knowledge application
	Muscle strength	Orientation to the person	General tasks &requirements: Undertaking a single task
	Muscle tone	Orientation to the own self	General tasks &requirements: Taking on multiple tasks
	Muscle endurance	Orientation to other persons	General tasks &requirements: Performing the daily routine
	Unintended movement responses		General tasks &requirements: Dealing with stress and other psychological demands
	Control of movements		Communication: Communicating as a receiver
	Unintended & random movements		Communication: Communicating as a sender
	Movement patterns		Communication: conversing & using communication devices & techniques
	Muscles & sensations related to movement		Mobility: Changing &maintaining body position
	Motor reflexes		Mobility: carrying, moving, &handling objects
			Mobility: Walking &moving around
			Mobility: Using transportation to get around
			Self-care: Washing oneself
			Self-care: Caring for body parts
			Self-care: Using the toilet
			Self-care: Dressing
			Self-care: Eating
			Self-care: Drinking
			Self-care: Taking care of your health
			Domestic life: Obtaining the necessities of life
			Domestic life: Household tasks
			Domestic life: Caring for household items & helping others
			Interpersonal interactions & relationships: General interpersonal relationships
			Interpersonal Interactions & Relationships: Special interpersonal relationships
			Significant areas of life: Education
			Significant areas of life: Work & employment
			Significant areas of life: Economic life
			Community, social & civic life: Community Life
			Community, social, & civic life: Recreation & leisure
			Community, social, & civic life: Religion & spirituality
			Community, social, & civic life: Human Rights
			Community, social, & civic life: Political life & citizenship
**Level**	No problem (none, absent, negligible…)		
	Moderate problem (medium, fair. . .)		
	Complete problem (total…)		

For the second experiment on neurological and perceptual disorders (neglect) the first chapter on “Mental functions” was used to define attributes. However, unlike the body functions not the whole chapter was used but only those aspects related to a neglect following stroke. On the first branching level this includes “Orientation functions” (b114) and “Visuospatial perception” (b1565). Within these, 6 factors are used to describe impairments due to neglect [19].

The third experiment is based in the activities and participation section of the ICF. All nine chapters and its respective first branching levels ("second level classifications") are used as attributes as all factors may influence the decision-making for rehabilitation and care for people experiencing impairments after stroke.

For all three experiments, the categories/criteria titled "otherwise specified" and "unspecified", respectively, were not considered for the decision model because no patient-relevant functions are categorized under these classifications that cannot also be subsumed under the other criteria. The descriptive framework of attributes and levels was developed with the MECE principles in mind in order to encompass all aspect while identifying the independent and important variables [22, 23].

After ethical approval, semi-structured interviews were conducted with patients and public representatives to further confirm this list of factors and provide feedback as to what extent the list is understandable and was appropriately re-worded from the clinical wording of the ICF to a patient-friendly expression. Interviewees indicated that the list of attributes had minimal overlap but was understandable. There were only a few minor suggestions to improve comprehensibility.

This validation activity led to minor amendments (e.g., wording) resulting into the final list of thirty-four attributes for the activity experiment (BWS case 1), twelve attributes for the arm paresis experiment (BWS case 2), six attributes for the neglect experiment (BWS case 3) (see Table 1 and Appendix A in S1 File for further description).

## 4. Methods

Stated preferences [24, 25] are an increasingly accepted approach to compare health technologies. The use of such choice experiments is rapidly increasing in medicine[26, 27], and professional societies have developed guidelines for the use of conjoint analysis in outcomes research [28, 29]. Stated preferences are an increasingly accepted method for generating preference data for ranking specific attributes or technologies within a disease class. Best-Worst Scaling (BWS) is a development of the classical Discrete Choice Experiment (DCE), which is becoming increasingly popular to elicit preferences in healthcare [28]. In contrast to DCE, where respondents choose only the best from a set of options, BWS requires respondents to identify the best and worst options in a choice scenario [30]. The main principle of the approach is that respondents define the extremes of a latent, subjective continuum. Essentially, they are asked to choose the pair that maximizes the value difference (on the scale of latent utility) between them [31]. The BWS method distinguishes three cases: the objective case (Case 1), the profile case (Case 2), and the multiprofile case (Case 3). Case 1 is used when the relative values of a set of objects or items (mostly called attributes) are of main interest [32]. The Case 2 scenario shows a single choice alternative, e.g., rehabilitation, defined by different attribute levels. The respondents are asked to choose the best (most important) and the worst (least important) attribute level of the given alternative. Case 3 is comparable to a classical DCE and provides multiple alternatives. However, next to the most preferred alternative respondents also choose the least preferred alternative. Supposing that there are only three choice alternatives in a choice scenario, a full preference ranking of the given alternatives can be determined [25, 33].

In this study, we will conduct three best–worst scaling (BWS) experiments, specifically one BWS experiment using each case to quantitatively assess the relative importance of body functions as well as impaired activities of daily living due to stroke long-term effects. A BWS object case is used for the activities because the respective activities and participation section of the ICF has no attribute and level structure [32]. These questions suit the purpose of this study as they are robust for scale-related biases, simplify ranking tasks for participants, and effectively discriminate between the ratings of different factors involved in complex decisions [34]. On the contrary, a certain attribute and level structure can be assumed for the body functions and the neurological and perceptual disorders. Hence more complex BWS cases can be applied. However, given the cognitive burden on respondents due to the high number of attributes we decided to use a profile case for the body functions while preferences on neglect will be assesses using a multiprofile case.

### The best–worst scaling experimental design

This quantitative elicitation is based on a choice experiment to analyze the final list attributes to understand what defines “value” from the perspective of patients. This protocol considers three experiments using a best-worst scaling that is based in the “stated preferences” tradition of economics evaluation.

This study will use an experimental-design software (Sawtooth Software’s Lighthouse [35]). An appropriate number of choice questions will be included in the experimental design so that we can estimate both main effects and possible selected interaction effects. This study involves an online survey designed as a best–worst scaling experiment. In all experiments an efficient experimental design will be used to reduce potential sample size and gain more insights on individual preferences of the targeted patient population. Due to the number of factors in the experiments, a fractional designs will be applied [36]. In such a design the selection of scenarios (i.e., combination of factors in the choice sets) is structured to generate the maximum amount of information. Fractional, efficient designs are characterized by (1) orthogonality (factors are shown and paired an approximately equal number of times), (2) minimal overlap (minimizing the number of times each factor appears within the same set across the design), (3) positional balance (factors appear approximately an equal number of times in each position), (4) connectivity (factors are directly or indirectly linked) and (5) stability (for each survey, four different versions of the questionnaire are used to increase variation). The interaction design might hold some level overlaps over both 2 and 3 alternatives. This results in easier choice tasks for the respondents. That is, respondents can concentrate on a subset of attributes and levels and are forced to make trade-offs for these remaining attributes. Moreover, the design of the BWS III will include dominant alternatives in a choice scenario with a high probability of being chosen for reasons of validity testing.

To realize a workable amount of choice sets, fractional, efficient designs are used which result in ten different versions of every survey to be generated for each of the three BWS experiments. This survey instrument provides a transparent, practical, and scientific approach to eliciting patient preferences in the context of patient-centered health technology assessments.

In this study, participants will be asked to complete a series of choice sets in which they have to choose the most and least important body functions addressed in neurorehabilitation or activities, respectively. For all three experiments the choice sets include a list of six items/attributes per set. All attributes are derived from the main list of functions and activities excerpted from the ICF. Within the experiments there are multiple tasks containing a set of factors in which the combination and ordering of factors differ.

### Questionnaire

An online survey was created using SurveyEngine, featuring a self-administered questionnaire. The survey structure is built upon the attributes and levels derived from the ICF, utilizing closed-ended questions. Participants are requested to provide online consent at the start of the questionnaire. To embrace a person-centered approach, the final survey instrument will include personalized details regarding attitudes and experiences, encompassing relevant clinical history, quality of life, and socio-demographic characteristics.

Within the elicitation task, ordinal responses will be elicited in the survey. A repeated survey is not planned (test-retest). In the decompositional BWS case III instrument, respondents will be asked to choose between 3 constructed hypothetical rehabilitation options including all attributes, with their levels varying in a sequence of choice sets [34]. Prior to answering the first task respondents will be made familiar with all the attribute and their associated levels. Also, a risk tutorial and an exercise for the actual choice task will be provided.

An estimated total of fifteen choice sets are used for each version of the questionnaire, with each choice set composed of six attributes from the respective decision model. The questionnaire versions are randomly allocated to participants except those participants experienced with arm paresis or neglect. These will be directed to the corresponding experiments. Furthermore, at the end of the survey, participants are asked to rate the difficulty of completing the questionnaire based on a Likert scale (1 = very easy, to 5 = very difficult).

Within the survey instrument, a "forced choice" will be integrated forcing the respondents to choose one of the presented therapy alternatives before being able to proceed with the experiment. As we do not plan to predict uptake, no opt-out option will be presented to capture more information on preferences with each choice. There will be no status quo alternative included in the choice scenarios [29]. The responding patients will be informed that the presented therapies might currently not be offered in this form, while the participating members of the public will be informed to assess what they would hypothetically choose if confronted with such a decision.

To reduce hypothetical bias and to engage respondents to give thoughtful answers, only one choice task per screen will be presented. A mapping between the instructions, choice profiles, and the response option will be provided. All elements of the choice task will be presented on the same screen; preferably without scrolling. The survey will be assessed on a variety of web browsers and mobile devices to ensure that choice tasks appear as intended.

After developing a draft survey instrument, the survey is being piloted prior to starting the process of data collection. Pre-test interviews are conducted to observe general reactions to the draft survey instrument and to determine which elements of the survey instrument require revision.

After finalizing the survey will be distributed via SurveyEngine to stroke patients, stroke care givers/families as well as representatives of the public. Additionally, a "planned stop" at an appropriate number of participants (N = 150) is set in the data collection of the survey. At this point, the data are analyzed for the first time to identify difficulties with the instrument or the design.

### Participants

The main target population are German stroke patients following acute hospital care (experienced, sample 1). To compare actual preferences with judgements we also aim to recruit a representative sample of the German general population confronted with making a hypothetical decision about (neuro-)rehabilitation (inexperienced, control group, sample 2). Hence, this study aims on recruiting two samples. All participants will be males or females aged 18 years or older, reside in Germany, able to read and understand German language and provide online informed consent.

Determining the appropriate sample size for DCE and BWS poses a challenge. Existing studies typically involve 100 and 300 respondents [37]. However, the minimum sample size varies based on several factors, such as question complexity, desired result precision, and the necessity for subgroup analyses [38]. Currently, there is no established guideline for the minimal sample size for BWS applications. Based on previous BWS studies, with sample sizes up to 800 participants [16, 36], this study aims to recruit approximately 1000 respondents from the general, ensuring at least 300 respondents participate in each of the three experiments (arm paresis, neglect and activities; sample 1).

Due to the seriousness of the disease, the patient sample size will be smaller (sample 2). The formula by Orme ($\frac{nta}{c}\geq 1000$) is used to calculate the best sample size [39]. The equation resulted in n = 72, given t = 14 choice tasks, a = 3 alternatives within BWS III and c = 3 levels each. Given this is the minimum required sample size, the plan is to recruit at least 100 patients [39].

The potential participants will be recruited by a vendor that sends invitations to members of their existing (panel) databases. The participants will have been already pre-profiled and have indicated they would like to contribute to research.

Next to this sample we will recruit a sample of stroke patients using advocacy groups/self-help groups as well as a national stroke foundation. As a final—and only if necessary–means of recruitment we will search social media groups or online forums for potential interest in contributing to the study. The inclusion criteria for the patient sample are consistent with those for the public (≥ 18 years old, residing in Germany, possessing the ability to read and comprehend German, and providing online informed consent). Additionally, patients must also have a self-indicated history of stroke. The study will not recruit minors.

### Ethics statement

The study is conducted according to the guidelines of the Declaration of Helsinki and approved by the Ethics Committee at University Greifswald. Online informed consent is obtained from all subjects involved in the study. At the beginning of the online survey, the participants must give their consent to the collection and further use of the data by clicking on the specific answer option. In the consent form, participants are also provided with further details of the survey, e.g., purpose, duration, benefits, and risks. Likewise, contact information is provided for any questions that may arise. Participants are free to stop their participation in the study at any time, by closing the respective webpage.

## 5. Data analysis

The models account for the panel structure of BWS data with the same respondent providing multiple outcomes for a sequence of different choice scenarios [40]. Upon reaching the target number of study respondents, analysis files will be constructed and data-quality checks as well as consistency tests on the choice data derived from the BWS questions will be performed. Subsequently, statistical analyses will be carried out on the BWS responses to quantify the relative importance of the study’s included attributes. The analysis will be conducted using Stata 15.1 [41].

The complexity of data-analysis tools, such as the specific logit-based model version, will be determined based on the data’s characteristics. Initial analysis will involve count analyses. The detailed analysis of BWS data will encompass evaluating validity issues among respondents and assessing the impact of these issues on average preference estimates. In this model, the dependent (response) variable will be the discrete treatment choice, while the independent (explanatory) variables include the attributes and levels describing the effects of neurorehabilitation treatment alternatives on boy function and activities.

Results from the logit-based models will constitute the primary outcome for this study, providing estimates of preferences for neurorehabilitation attributes. This will include conditional as well as mixed logit models. Second, an exploratory latent class analysis will be conducted to investigate potential variations in preferences based on patient characteristics, including but not limited to sociodemographic factors, attitudes, and experiences. If statistically significant differences in preferences are found among subgroups, subgroup-specific preference information will be reported [37, 42].

## 6. Expected results

To our knowledge, this is the first study assessing the relative importance of (sub-)chapters of the ICF against the background of decision-making for neurorehabilitation following stroke. When designing and evaluating rehabilitation care, it is important to understand the preferences of patients on how they value various factors, to be able to optimize patient-centered care [8, 16].

Results are expected by the end of May 2022. The expected results can inform value judgements of clinical, policy and regulatory decision makers in approval, reimbursement, pricing, and utilization decisions in terms of robot assisted neurorehabilitation. Findings will inform about the weighting of two subchapters as well as the activity section of the ICF among patients and public representatives in Germany, confronted with the actual decision or a hypothetical judgment. The research will add to the current literature on clinical-decision-making in stroke rehabilitation. The findings will inform future research and consensus activities to derive a more patient-centered classification of impairments and treatment goals to guide decision-makers in stroke units to prioritize the factors for deciding rehabilitation for stroke patients.

## 7. Discussion

Clinical studies in neurorehabilitation measure clinical parameters and indicators that are an expression of arm mobility or visuospatial perception. These are the result of certain bodily functions. However, it is unclear whether measurable functions or improvement in function have an impact on the assessable (patient) benefit. If the fulfillment or improvement of functions has effects on patients’ activities and assuming these activities have measurable influences in terms of patients’ (health-related) quality of life raises the question how the value of functions can be measured?

After assessing the quality of clinical evidence, clinicians and decision makers often must make complex decisions about access, reimbursement, and use. These decisions require nonclinical value judgments about whether the benefits of robot-assisted therapies justify the associated expense. Such societal value judgments are traditionally made by experts. However, because patients are the ones who ultimately experience the positive and negative outcomes of treatment, most decision makers agree that decisions related to new therapeutic options should be patient-centered and reflect patient values.

The value of an intervention depends on what clinical effects can be achieved, how patients evaluate them for themselves, and what effort patients associate with an intervention. The acceptance of stroke patients is an essential prerequisite for achieving clinical effects. In addition, it is important to find out which characteristics are an incentive for the patients to participate and which ones may be an effort and thus lead to the rejection of an intervention. This is where health preference research comes in, providing information about patient value judgments relevant to decision-making.

Due to the enormous importance of preferences, methods for their measurement are increasingly used in health services research and in health economics. However, in the search for the best possible solution, decision-makers almost always must weigh several attributes of an alternative against each other, which requires so-called multi-attribute evaluation methods.

The BWS method used has several strengths. The main strength of BWS is the better information yield by inducing respondents to make two statements rather than just one. The preference structure of the respondents can thus be determined more precisely or with equal precision, but with a smaller sample size. BWS presents an alternative preference elicitation method that can resolve some of the limitations of the traditional DCE technique and especially manage a high number of attributes [43]. In addition, repeated BWS questions allow to derive complete rather than partial rankings by gradually excluding alternatives previously identified as "best" or "worst." Reduced cognitive load on subjects is cited as another advantage of BWS [34]. It seems to be easier for the subjects to determine the two respective extreme points on their utility scale than to select the most preferred alternative between two or more alternatives with many attributes in complex decision scenarios or even to determine a complete ranking [44]. In addition to advantages, BWS also has weaknesses, which ultimately stem from the simple fact that even in experiments additional information can only be had at increased cost. Thus, for the subjects, the time required for the choice decision increases [45, 46].

## 8. Limitations

This study has potential limitations. First, there is a certain risk of an interviewer bias within the results of the pretest-interviews. Since the participants were interviewed in person and/or virtually, socially desirable statements cannot be avoided. In addition, members of the research consortium were always present in the room with the patients if the interviews were conducted online by the responsible researcher. Even if these members are not directly involved in these preference studies but only the clinical study, influences on the participants’ answers cannot be eliminated.

Second, as the factors and attributes used in the BWS experiments cover only the chapters of the ICF related to arm paresis, neglect, and activities of daily living. Hence, the findings of this study may not be generalizable to other chapters of the ICF or the ICF in general. Third, there is a potential risk for certain groups of patients and or the public to be under- or over-represented in the survey due to actual non-response. Additionally, the number of participants may not be equally distributed among the three experiments. Hence, the overall ranking of factors may over-represent specific subgroups. However, latent class analyses will outline to what extent there are group differences, and heterogeneities [42].

Fourth, the recruitment of participants uses a vendor with an online panel. It could be argued that this leads to a certain selectin bias as only this with internet access may participate. Moreover panelists might more likely belong to a homogenous educational class and it has to be mentioned that they earn money via panels when completing numerous surveys [47]. Fifth, given the severity of the disease and the potential impairments following a stroke, possible barriers to participation due to physical conditions may apply. However, persons with certain impairments (e.g., visual impairments) are not excluded. The vendor that will recruit patients /public is open to everyone. Hence, it is assumed that participants with impairments have the necessary technical equipment to enable them to participate in the online study (e.g., reading software, magnification) or have the assistance of caregivers in answering the questions. Sixth, participants may not be familiar with the BWS process since it is a relatively new elicitation method, which could lead to non-response response bias due to dropping out of the survey. Seventh, this study aims to understand the preferences of patient and public representatives confronted with decision-making for robot-assisted neurorehabilitation after stroke. However, considering shared decision-making, future studies may analyze preferences from the clinician perspective.

Finally, the survey is being conducted during the Covid 19 pandemic. It is entirely possible that the results would have been different if the survey had been conducted before or after the outbreak and that patient preferences for robotic and/or non-personal therapies are significantly higher during the pandemic than without pandemic. With the coronavirus outbreak and the concomitant increase in digital counseling and care, a differential view and choice of individuals is also conceivable or even assumed also for robotic assistance. Avoiding the risk of infection through contact in waiting rooms, therapists’ offices or public transport could be crucial in this regard. However, healthcare also thrives on the personal caregiver-patient relationship. Moreover, the discussion about data protection could also be perceived negatively and increase the unpleasant feeling of being constantly monitored. Another study under different circumstances could shed light on this. Finally, the pandemic could function as a digital health accelerator for changing perceptions and acceptance of digital or robotic health solutions.

## Supporting information

S1 File Description of attributes for BWS I (Activity). (DOCX)

S2 File Description of attributes for BWS II (Body functions). (DOCX)

S3 File Description of attributes for BWS III (Neglect). (DOCX)
