# Supplementary material for: Which body functions and activities matter for stroke patients? Study protocol for best–Worst scalings to value core elements of the International Classification of Functioning, Disability and Health
Source: PLoS One. 2023 Dec 7;18(12):e0295267. doi: 10.1371/journal.pone.0295267 (PMC10703233; doi:10.1371/journal.pone.0295267)
Supplement: S1 File — (DOCX) [file pone.0295267.s001.docx]

## Appendix A. Description of Factors

### Table A1. Description of factors: Body function (BWS case II)

|  | **Attribute** | **Easy-to-understand description** |
| --- | --- | --- |
| **Functions of the joints and bones** | **Mobility of joint functions** | Describes the range of motion and ease of movement. This includes the suppleness of joints, such as the shoulder, elbow, wrist, and fingers. Dysfunctions include over-movement of joints, joint stiffness, or even joint inflammation. |
|  | **Stability of joint functions** | The basis for pain-free movement is the strength/ robustness of joints. This refers to the reliability of joints in the arm and hand, i.e., shoulder, wrist, elbow. |
|  | **Mobility of bone functions** | Describes the range of motion and ease of movement from the scapula and carpal bones. |
| **Muscle functions** | **Muscle power functions** | Describes the power with which muscles contract. This includes the strength of the muscles in the arm and hand. Possible dysfunctions include muscle weakness or muscle paralysis. |
|  | **Muscle tone functions** | A certain muscle tension at rest is the basis for all movements. If this tension is not present or is present incorrectly, functional disorders occur. These include too low or too high muscle tension or spasticity (permanent hardening and stiffening. These are associated with limitations in mobility). |
|  | **Muscle endurance functions** | Describes the maintenance of muscle strength and tension in the muscles of the arm or hand over a period. This is important to perform prolonged movements. |
| **Movement functions** | **Involuntary movement functions** | Describes the random/reflex contraction of large muscles or the whole body. This is triggered by body posture or balance and startle reactions. Includes reactions to changes in position (e.g., turning over), straightening/standing up, as well as balance reactions, support reactions, defensive reactions, e.g., stumbling. |
|  | **Control of voluntary movement functions** | Describes the control and coordination of conscious/intentional movements. Includes coordination of movements, support functions of arms, and eye-hand coordination, e.g., writing, inserting keys into a lock. |
|  | **Involuntary movement reaction functions** | Describes involuntary, random contraction of muscles. Includes dysfunctions such as tremors, "nervous twitching" (tics), vocal tic (involuntary calling out of words, sounds), teeth grinding, repetitive movements, or spontaneous movements such as rocking back and forth, head nodding, or head bobbing. |
|  | **Gait pattern functions** | Describes the movements involved in walking, running, or other movements of the body. Includes dysfunctions such as walking with spasticity/paralysis, limping, and a stiff gait. |
|  | **Sensations related to muscles and movement functions** | Describes sensations associated with muscles in the arm and hand and movements. These include muscle stiffness and tightness, muscle spasms, or a feeling of heaviness in the muscles. |
|  | **Motor reflex functions** | Describe an involuntary contraction of the muscles. These include stretch reflexes, reflexes to harmful external influences, and protective reflexes or various intrinsic reflexes in the arm. |
