# Supplementary material for: Which body functions and activities matter for stroke patients? Study protocol for best–Worst scalings to value core elements of the International Classification of Functioning, Disability and Health
Source: PLoS One. 2023 Dec 7;18(12):e0295267. doi: 10.1371/journal.pone.0295267 (PMC10703233; doi:10.1371/journal.pone.0295267)
Supplement: S2 File — (DOCX) [file pone.0295267.s002.docx]

## Appendix A. Description of Factors

### Table A2. Description of factors: neurological and perceptual disorder/ neglect (BWS case III)

|  | **Attribute** | **Easy-to-understand description** |
| --- | --- | --- |
| **Visuospatial perception** | **Visuospatial perception** | Mental function involved in distinguishing by sight the relative position of objects in the environment or in relation to oneself, e.g., distance to objects, walls, doors. |
| **Orientation functions** | **Orientation to time** | Mental functions that produce awareness of day, date, month, and year. |
|  | **Orientation to place** | Mental functions that produce awareness of one's location, such as one's immediate surroundings, one's town or country. |
|  | **Orientation to person** | Mental functions that produce awareness of one's own identity and of individuals in the immediate environment |
|  | **Orientation to self** | Mental functions that produce awareness of one's own identity. |
|  | **Orientation to others** | Mental functions that produce awareness of the identity of other individuals in one's immediate environment. |
