# Supplementary material for: Which body functions and activities matter for stroke patients? Study protocol for best–Worst scalings to value core elements of the International Classification of Functioning, Disability and Health
Source: PLoS One. 2023 Dec 7;18(12):e0295267. doi: 10.1371/journal.pone.0295267 (PMC10703233; doi:10.1371/journal.pone.0295267)
Supplement: S3 File — (DOCX) [file pone.0295267.s003.docx]

## Appendix A. Description of Factors

### Table A3. Description of factors: Activities of daily living (BWS case I)

|  | **Attribute** | **Easy-to-understand description** |
| --- | --- | --- |
| **Learning & knowledge application** | **Conscious sensory perceptions** | Describes activities such as   - Watching, (perceiving visual stimuli) - listening (perceiving acoustic stimuli) - - Other conscious sensory perceptions (touching and feeling surfaces, tasting, or smelling something). |
|  | **Elementary learning** | Describes activities such as.   - imitating/imitating - practicing - learning to read - learning to write - learning arithmetic - acquiring skills |
|  | **Knowledge application** | Describes activities such as   - focusing attention - thinking - reading - writing - arithmetic - solving problems - making decisions |
| **General tasks & requirements** | **Perform single task** | Perform coordinated actions on a single task. This includes organizing time, space, and materials for the task, determining the steps of performance, performing the task, and completing the task. |
|  | **Perform multiple tasks** | Perform coordinated actions as components of a comprehensive task in sequential steps or simultaneously. |
|  | **Perform daily routine** | Perform systematic actions to plan and manage the demands of daily duties, such as scheduling time and making the daily plan for various activities |
|  | **Deal with stress and other psychological demands** | Perform systematic actions to accomplish tasks that involve special responsibilities and are associated with stress, disruption, and crisis situations. These may include driving a vehicle in heavy traffic or caring for many children |
| **Communication** | **Communicate as receiver** | Describes activities such as.   - communicating as a recipient of spoken messages - communicating as a recipient of non-verbal messages (gestures, symbols, and drawings). - communicating as a recipient of sign language messages - communicating as a recipient of written messages |
|  | **Communicating as a sender** | Describes activities such as.   - speaking - producing non-verbal messages (gestures, symbols, and drawings) - expressing messages in sign language - writing messages |
|  | **Conversation and use of communication devices and techniques** | Describes activities such as.   - conversation/conversations (with one or more known or unfamiliar people) - discussion (with one or more known or unfamiliar people) - using communication devices and techniques, e.g., internet, telephone, cell phone |
| **Mobility** | **Change and maintain body position** | Describes activities such as   - changing a body position - remaining in one body position - shifting |
|  | **Carry, move, and handle objects** | Describes activities such as   - lifting and carrying objects - moving objects with legs - fine motor hand use - hand and arm use, e.g., throwing or catching |
|  | **Walk and move around** | Describes activities such as.   - walking - moving around in other ways, e.g., crawling, climbing, running, jogging, jumping, swimming - moving around in different environments, e.g., in different places and situations/ buildings - moving around using devices/equipment, e.g., using a wheelchair or walker |
|  | **Moving around with means of transportation** | Describes activities such as.   - using a means of transportation, e.g., cab, train, streetcar, subway, ship, or airplane - driving a vehicle, e.g., car, bicycle, boat, or carriage - riding animals for transportation, e.g., horse |
| **Self-care** | **Washing yourself** | Wash and dry the body or parts of the body (hands, feet, face, hair). |
|  | **Caring for body parts** | Taking care of one's body parts such as skin, face, teeth, scalp, nails, e.g., applying cream, combing. |
|  | **Using the toilet** | Performing human excretions (urine, stool) as well as cleaning oneself afterwards. |
|  | **Dressing** | Put on and take off clothes and shoes according to social conditions and climatic requirements. |
|  | **Eating** | Bringing food to the mouth and eating it, cutting food into pieces, opening bottles and cans, using cutlery. |
|  | **Drinking** | Pick up a container with a drink, bring it to the mouth and drink the contents, or drink running water such as from a faucet or spring. |
|  | **Take care of own health** | Ensure health and physical and mental well-being, e.g., eat a balanced diet and be physically active, keep warm, avoid illness and injury, ensure immunizations and regular medical checkups. |
| **Domestic life** | **Procurement of necessities of life** | Describes activities such as.   - procuring, buying, renting living space (house/apartment) - procure goods and services of daily use, e.g., food, beverages, clothing, and household items. |
|  | **Household tasks** | Describes activities such as.   - preparing meals (food and beverages) - do household chores, e.g., wash clothes, dispose of garbage, iron, clean house |
|  | **Maintaining household items and helping others** | Describes activities such as.   - maintaining household items, e.g., car, bicycle, plants, renovating, caring for house animals - helping other people |
| **Interpersonal interactions & relationships** | **General interpersonal relationships** | Describes activities such as.   - elementary interpersonal activities such as showing consideration and appreciation or responding to feelings. - complex interpersonal relationships, such as maintaining exchanges with others, controlling, and managing emotions and aggression. |
|  | **Special interpersonal relationships** | Describes activities such as.   - interacting with strangers, e.g., asking directions or buying groceries - formal relationships, e.g., with employers or service providers, such as doctors/therapists - casual social relationships, e.g., having casual relationships with people who live in the same community or house, acquaintances, or friendships - family relationships - intimate relationships |
| **Significant areas of life** | **Education** | Describes activities such as.   - informal education/training: learning crafts and other skills at home from parents or family members. - preschool education, i.e., preparing a child for school - school education - theoretical vocational education, i.e., vocational school - higher education and training, e.g., universities, colleges, and technical schools. |
|  | **Work and employment** | Describes activities such as.   - preparation for gainful employment, such as apprenticeships, internships, and training that accompanies education and training - getting, keeping, and finishing a job - paid activity, such as being employed, working full or part time, or self-employed - unpaid activity, e.g., volunteering in clubs, for the community, for religious groups, or in the home environment |
|  | **Economic Life** | Describes activities such as.   - elementary economic transactions, such as using money to buy food or exchange goods and services or save money - comprehensive economic transactions, such as buying a business, keeping a bank account, or trading commodities - economic autonomy: having control over economic resources (money, property). |
| **Community, social & civic life** | **Community life** | Engage in aspects of community social life, such as participating in charities or social organizations. |
|  | **Recreation and leisure** | Engage in games, recreational or leisure activities, such as physical fitness, relaxation, entertainment, or culture. These include visiting art galleries, museums, movies, or theaters; doing handicrafts and pursuing hobbies; reading; playing musical instruments; traveling. |
|  | **Religion and spirituality** | Engaging in religious and spiritual activities and forming relationships with a divine power, such as participating in devotions in a church, temple, mosque, or synagogue, praying and singing for religious reasons. |
|  | **Human Rights** | To enjoy the nationally and internationally recognized rights granted to human beings solely by virtue of being human, such as the human rights of the United Nations Declaration of Human Rights (1948), the right to self-determination and autonomy, and the right to determine one's own destiny. |
|  | **Political life and citizenship** | Participate as a citizen in social, political, and governmental life and pursue the rights and responsibilities that go with it, such as exercising the right to vote, running for political office, forming political associations |
